# Supplementary material for: Epidemiological observations and management challenges in extrapedal mycetoma: A three-decade review of 420 cases
Source: PLoS Negl Trop Dis. 2024 May 10;18(5):e0011841. doi: 10.1371/journal.pntd.0011841 (PMC11111073; doi:10.1371/journal.pntd.0011841)
Supplement: Attachment — Submitted filename: Mycetoma extrapedal 2024.docx [file pntd.0011841.s002.docx]

Dear Sir,

Thank you so much for sending the manuscript “Epidemiological Observations and Management Challenges in Extrapedal Mycetoma: A Three-Decade Review of 420 Cases” for the review.

It’s a well written paper, should be acceptable but only after minor corrections and changes.

My observations are as follows.

- Line 99: please elaborate the word “workers.” It is important to know the nature of jobs these workers, we know that acquisition of mycetoma is linked with nature the of job
- **References**
  - In the manuscript, reference style should remain as per journal guidelines, not like this 6,7,8 (line # 92). Need to be corrected at many places.
  - Italicize name of microorganisms in the references (for example, line # 607, 611, 664 and others)
- Please correct spelling line# 640 “extrapodal”
- **Numbers and percentage**: Authors need to recalculate numbers and percentages altogether, there are multiple mistakes in text and tables (Table1 “occupation”: it is neither 420 nor 100%). Probably table 2(first heading “size”: total number and percentage are not correct).
- Line # 157, 161, 265 and 286 need corrections.
- Table 2: not labelled properly.
- Line # 363-366: Many information’s are given but are not in flow. Please rewrite these lines
- Line 376: S should be large for *Staphylococcus aureus*

All the best

Dr. Afia Zafar,

Professor and Consultant Microbiologist

Department of Pathology and Laboratory Medicine

Aga Khan University, Karachi, Pakistan
