# Supplementary material for: Epidemiological observations and management challenges in extrapedal mycetoma: A three-decade review of 420 cases
Source: PLoS Negl Trop Dis. 2024 May 10;18(5):e0011841. doi: 10.1371/journal.pntd.0011841 (PMC11111073; doi:10.1371/journal.pntd.0011841)
Supplement: Attachment — Submitted filename: Response to Reviewers.docx [file pntd.0011841.s003.docx]

**Response to Reviewers**

**Dear Editor and Reviewers,**

Thank you for your thoughtful comments on our manuscript, "**Epidemiological Observations and Management Challenges in Extrapedal Mycetoma: A Three-Decade Review of 420 Cases."**

Our responses to the editor's and reviewer's comments are below. Please do not hesitate to let us know if our responses need clarification or if the revised manuscript requires further editing.

Sincerely,

Prof Fahal

**The editor's comments**

1) Please be so kind and label your Tables in numerical order. (For ex: Table1, Table2, Table3, table4). Currently two tables are labeled as Table 1 ( The study population demographic characteristics,The clinical presentation of the study population) and Table 3 was not labeled.

**Kindly note we addressed this request twice in the already uploaded clean manuscript and the manuscript with the changes highlighted, which were uploaded into the system on 22 February 2024 and once again uploaded on 29 February. Kindly clarify for us the problem**

line no 296; we refer to Table 3 in the no mass detected row, as the masses were removed elsewhere and were detected by ultrasound only

| Small | 45 | 10.71% |
| --- | --- | --- |
| Medium | 125 | 29.76% |
| Massive | 185 | 44.04% |
| No mass detected (Post-operative) | 48 | 11.42% |
| Missing | 17 | 04.04% |

Here, once again, the tables labels

The tables labels were done as follows:

Table 1: The study population demographic characteristics line 175

Table 2. The clinical presentation of the study population. Line 235

Table ‎3. The clinical examination findings among the study population. Line 295

Table 4: The patients' treatment outcomes distribution according to the mycetoma type among patients with regular follow-up. Lines 389-390

2) Thank you for stating" Please be informed that ethical clearance was secured from the Institutional Board Review of Soba University Hospital, University of Khartoum, under the reference number SUH/IRB/2022/34. In relation to the investigations conducted for confirmation of the diagnosis, no consents were acquired, as they were part of the Mycetoma Research Center patients' management guidelines.

Please be so kind and update the details in the manuscript file.

The ethical clearance number was added at lines 142-143

3) "We note that there is identifying data in the Supporting Information file < data- revised exam finding 15112022.xls>. Due to the inclusion of these potentially identifying data, please remove this file from your file inventory. Prior to sharing human research participant data, authors should consult with an ethics committee to ensure data are shared in accordance with participant consent and all applicable local laws.
 
 Data sharing should never compromise participant privacy. It is therefore not appropriate to publicly share personally identifiable data on human research participants. The following are examples of data that should not be shared:
 
 - Name, initials, physical address
 - Ages more specific than whole numbers
 - Internet protocol (IP) address
 - Specific dates (birth dates, death dates, examination dates, etc.)
 - Contact information such as phone number or email address
 - Location data
 - ID numbers that seem specific (long numbers, include initials, titled "Hospital ID") rather than random (small numbers in numerical order)
 
 Data that are not directly identifying may also be inappropriate to share, as in combination they can become identifying. For example, data collected from a small group of participants, vulnerable populations, or private groups should not be shared if they involve indirect identifiers (such as sex, ethnicity, location, etc.) that may risk the identification of study participants.
 
Additional guidance on preparing raw data for publication can be found in our Data Policy:  [insert relevant link]  
<https://emea01.safelinks.protection.outlook.com/?url=https%3A%2F%2Fjournals.plos.org%2Fplosntds%2Fs%2Fdata-availability%23loc-human-research-participant-data-and-other-sensitive-data&data=05%7C02%7C%7C2fa228100c9e44cde97708dc343ed9b2%7C84df9e7fe9f640afb435aaaaaaaaaaaa%7C1%7C0%7C638442691748788104%7CUnknown%7CTWFpbGZsb3d8eyJWIjoiMC4wLjAwMDAiLCJQIjoiV2luMzIiLCJBTiI6Ik1haWwiLCJXVCI6Mn0%3D%7C0%7C%7C%7C&sdata=JxbnVy67ufSG0%2FoTStLjm2bgHfdqZ4rK%2Bs2U0hKvPUs%3D&reserved=0> 
   
You may also find guidance in the following article: <https://emea01.safelinks.protection.outlook.com/?url=http%3A%2F%2Fwww.bmj.com%2Fcontent%2F340%2Fbmj.c181.long&data=05%7C02%7C%7C2fa228100c9e44cde97708dc343ed9b2%7C84df9e7fe9f640afb435aaaaaaaaaaaa%7C1%7C0%7C638442691748796775%7CUnknown%7CTWFpbGZsb3d8eyJWIjoiMC4wLjAwMDAiLCJQIjoiV2luMzIiLCJBTiI6Ik1haWwiLCJXVCI6Mn0%3D%7C0%7C%7C%7C&sdata=wrM6TkIJF1Hg%2B%2B62QtuEfF%2Bq4%2BhreKqZRFT3Tke7gtc%3D&reserved=0>.
 
 Please remove or anonymize all personal information, ensure that the data shared are in accordance with participant consent, and re-upload a fully anonymized data set. Please note that spreadsheet columns with personal information must be removed and not hidden as all hidden columns will appear in the published file.

All personal information was removed.

4) "Some material included in your submission may be copyrighted. According to PLOS's copyright policy, authors who use figures or other material (e.g., graphics, clipart, maps) from another author or copyright holder must demonstrate or obtain permission to publish this material under the Creative Commons Attribution 4.0 International (CC BY 4.0) License used by PLOS journals. Please closely review the details of PLOS's copyright requirements here: PLOS Licenses and Copyright. If you need to request permissions from a copyright holder, you may use PLOS's Copyright Content Permission form.

Please respond directly to this email and provide any known details concerning your material's license terms and permissions required for reuse, even if you have not yet obtained copyright permissions or are unsure of your material's copyright compatibility. Once you have responded and addressed all other outstanding technical requirements, you may resubmit your manuscript within Editorial Manager.

Potential Copyright Issues:

1) Thank you for stating: Figures 3 and 4 constitute portions of the MRC photography repository.

Please provide written permission from the copyright holder to publish the photo(s) under our CC BY 4.0 license.
written permission from the Mycetoma Research Center is provided to publish the photos 3,4 under the Creative Commons Attribution 4.0 International (CC BY 4.0) License used by PLOS journals. – it is uploaded, and CCBY 4.0 is signed and loaded

2) [Figure 2]: Please confirm whether you drew the images / clip-art within the figure panels by hand. If you did not draw the images, please provide (a) a link to the source of the images or icons and their license / terms of use; or (b) written permission from the copyright holder to publish the images or icons under our CC BY 4.0 license. Alternatively, you may replace the images with open source alternatives. See these open source resources you may use to replace images / clip-art:
- <https://emea01.safelinks.protection.outlook.com/?url=https%3A%2F%2Fcommons.wikimedia.org%2F&data=05%7C02%7C%7C2fa228100c9e44cde97708dc343ed9b2%7C84df9e7fe9f640afb435aaaaaaaaaaaa%7C1%7C0%7C638442691748801688%7CUnknown%7CTWFpbGZsb3d8eyJWIjoiMC4wLjAwMDAiLCJQIjoiV2luMzIiLCJBTiI6Ik1haWwiLCJXVCI6Mn0%3D%7C0%7C%7C%7C&sdata=YpVX3gWu0m6xtTLFykl4qvzHz7e%2Ff9EI9%2FpH4Lpng28%3D&reserved=0>
- <https://emea01.safelinks.protection.outlook.com/?url=https%3A%2F%2Fopenclipart.org%2F&data=05%7C02%7C%7C2fa228100c9e44cde97708dc343ed9b2%7C84df9e7fe9f640afb435aaaaaaaaaaaa%7C1%7C0%7C638442691748806533%7CUnknown%7CTWFpbGZsb3d8eyJWIjoiMC4wLjAwMDAiLCJQIjoiV2luMzIiLCJBTiI6Ik1haWwiLCJXVCI6Mn0%3D%7C0%7C%7C%7C&sdata=kbRX4xCxu35dO7OHzV6OfA2Jtps29EyctyCDFKYtbjs%3D&reserved=0>

Figure 2 was created by one of the authors.

2) [Figure1]: Thank you for stating" The base map shapefile, onto which data has been plotted, originates from ArcGIS, version 3"

   Please provide a DIRECT LINK to the base layer of the map (i.e., the country or region border shape) and ensure this is also included in the figure legend;

   The link to the base layer of the map (i.e., the country or region border shape) is included in the figure and its legend. Lines 713-714

<https://www.arcgis.com/home/item.html?id=7ae5637c804b4b6e80abb85d0ca2c26c>

6) Thank you for stating" The diagnosis of mycetoma was confirmed by careful interview, meticulous  clinical examinations, and specific investigations, including fine needle aspiration for cytology (FNAC), histopathological examination of surgical biopsies, grains culture and molecular techniques to 134 identify the microorganism  to the species level".

Please include a statement that formal consent was obtained (must state whether verbal/written) OR the reason consent was not obtained (e.g. anonymity). NOTE: If child participants, the statement must declare that formal consent was obtained from the parent/guardian/head of household"

These procedures are part of the routine investigation done at the centre to confirm the diagnosis.

**The Reviewer**

Dear Sir,

Thank you so much for sending the manuscript “Epidemiological Observations and Management Challenges in Extrapedal Mycetoma: A Three-Decade Review of 420 Cases” for the review.

It’s a well written paper, should be acceptable but only after minor corrections and changes.

My observations are as follows.

- Line 99: please elaborate the word “workers.” It is important to know the nature of jobs these workers, we know that acquisition of mycetoma is linked with nature the of job

They are manual workers, which is corrected in line 98

- **References**
  - In the manuscript, reference style should remain as per journal guidelines, not like this 6,7,8 (line # 92). Need to be corrected at many places.

That is checked and corrected.

- Italicize name of microorganisms in the references (for example, line # 607, 611, 664 and others)

Corrected

- Please correct spelling line# 640 “extrapodal”

Corrected

- **Numbers and percentage**: Authors need to recalculate numbers and percentages altogether, there are multiple mistakes in text and tables (Table1 “occupation”: it is neither 420 nor 100%).

Corrected in line 175

- Probably table 2(first heading “size”: total number and percentage are not correct).

Corrected

- Line # 157, 161, 265 and 286 need corrections.

Corrected

- Table 2: not labelled properly.

Done line 235

- Line # 363-366: Many information’s are given but are not in flow. Please rewrite these lines

Done

- Line 376: S should be large for *Staphylococcus aureus*

Corrected in line 386

All the best

Dr. Afia Zafar,

Professor and Consultant Microbiologist

Department of Pathology and Laboratory Medicine

Aga Khan University, Karachi, Pakistan
